# Electrically and Thermally Triggered Three-Dimensional Graphene-Foam-Reinforced Shape Memory Epoxy Composites

**DOI:** 10.3390/polym15132903

**Published:** 2023-06-30

**Authors:** Adeyinka Idowu, Tony Thomas, Jenniffer Bustillos, Benjamin Boesl, Arvind Agarwal

**Affiliations:** Plasma Forming Laboratory, Department of Mechanical and Materials Engineering, Florida International University, Miami, FL 33174, USA; aidow002@fiu.edu (A.I.); tonthoma@fiu.edu (T.T.); jab925@cornell.edu (J.B.); bboesl@fiu.edu (B.B.)

**Keywords:** graphene foam, shape memory polymer epoxy, glass transition temperature, dual triggering, shape recovery, self-healing

## Abstract

Shape memory polymer (SMP) epoxy composites have attracted significant attention due to their easy processing, lightweight nature, and ability to recover strain. However, their limited recovery rate and inferior mechanical properties have hindered their functional applications. This research explores the potential of three-dimensional (3D) graphene foam (GrF) as a highly efficient reinforcement for SMP epoxy composites. We demonstrated that the incorporation of a mere 0.13 wt.% GrF into mold-cast SMP epoxy leads to a 19% increase in the glass transition temperature (T_g_). To elucidate the reinforcing mechanism, we fabricated and extensively analyzed composites with varying weight percentages of GrF. The GrF-based SMP epoxy composite exhibits a 57% increase in thermal conductivity, measuring 0.296 W mK^−1^ at 70 °C, due to the interconnected 3D graphene network within the matrix. Notably, this composite also demonstrates remarkable electrical conductivity, making it suitable for dual-triggering applications. The GrF-SMP epoxy composite achieves a maximum shape recovery ratio and a significant 23% improvement in the recovery rate, effectively addressing the issue of slow recovery associated with SMPs. We investigated the effect of switching temperatures on the shape recovery rate. We identified the optimal triggering temperature to initiate shape recovery for epoxy SMP and GrF-epoxy SMP as thermal energy equivalent to T_g_ + 20 °C. Additionally, we fabricated a bird-shaped composite using GrF reinforcement, which showcases self-healing capabilities through the crack opening and closure and serves as a tangible demonstration of the transformative potential of the composite. These GrF-epoxy SMP composites, responsive to stimuli, hold immense promise for diverse applications, such as mechanical systems, wearable sensors, morphing wings, foldable robots, and antennas.

## 1. Introduction

The increasing need for smart devices for biomedical [1], electronic [2], automotive, and aerospace [3] applications has significantly influenced shape memory materials in recent years [4]. Among the types of shape memory materials, shape memory metallic alloys (SMA) and shape memory polymers (SMP) are the most widely used. However, SMPs have gained more attention due to their facile processing [5], low cost [6], light weight, and high recoverable strain [7], up to 700% compared to SMA. SMP are conventional polymers engineered to exhibit shape memory behavior. The molecular structural design consists of net points and switching segments responsible for displaying the shape memory effect (SME) in polymers. SME is the ability of SMP to retain its deformed temporary shape and recover to its permanent form under exposure to external stimuli, such as heat [8], electricity [9], light [10], magnetic [11], pH, water/solvent ions [12], and ultrasound [13]. Epoxy SMP is a typical example of a thermally stimulated polymer, which can demonstrate SME. Fabrication process of the SMP components partly influences the SME. Mold casting [14], injection molding [15], thermoplastic extrusion [16], and additive manufacturing [17] are used in the fabrication of SMP parts. It is also worth noting that some advanced techniques, such as 4D printing [18,19], are employed to fabricate self-assembling SMP parts with unique architecture. The choice of epoxy SMP as a material is due to low shrinkage during curing, versatility in chemical structures, and long environmental durability [20,21]. Epoxy SMP is composed of aromatic and aliphatic epoxies. These epoxies provide the capability to memorize the permanent shape and change the temporary shape when triggered under the influence of heat. However, its low elastic modulus (1.4–2.5 GPa) [22,23], low thermal conduction (<0.3 W/mK) [24], low recovery stress (1–3 GPa) [25], and slow recovery time are still a concern for the employment of epoxy SMP in actuated devices. Its low intrinsic stiffness accounts for the low recovery stress generated during shape recovery. Poor heat transfer also leads to slow thermal diffusion through the epoxy SMP. This translates to a longer recovery period for the epoxy SMP to attain its permanent shape. However, research efforts have been geared towards addressing the drawbacks of epoxy SMP using conventional inorganic fillers [7]. These challenges can be further resolved by reinforcing epoxy SMP with graphene-based fillers to boost the shape memory performance of epoxy SMP [26,27,28,29,30].

Graphene, a two-dimensional (2D) monolayer of sp^2^ bonded carbon atoms, is a good filler in polymers. Its remarkable electrical [31], thermal [32], and mechanical properties [33,34] have facilitated performance improvement in polymer composites. Similarly, graphene addition in SMP can enhance shape memory properties [35]. This is because it can provide the heat conduction needed to kickstart SME in SMP. Graphene’s electro-thermal property offers SMEs electrical stimulation, an attractive SMP stimulus source [36,37]. The delocalized π electrons within the conduction band account for the electron flow in graphene sheets. In contrast, anisotropic solid bonding and the low mass of carbon atoms can be attributed to the easy phonon flow within the SMP.

A few studies have examined graphene’s influence on the shape memory behavior of epoxy SMPs. Wang et al. revealed that the shape recovery ratio and shape memory H-EP recovery speed increased as the graphene content increased from 1 wt.% to 3 wt.% [38]. Another investigation showed that reducing the cross-linking density of graphene with epoxy SMP could improve the shape memory recovery behavior [39]. Despite the advances in the shape memory behavior of 2D graphene-reinforced SMPs, graphene addition challenges still exist. This includes weak interfacial interaction and graphene sheet aggregation, which impede the benefits of graphene addition [40,41]. Yonessi et al. modified the surface to improve the dispersion and create a better interface for the shape memory behavior of SMP [35]. Wang et al. highlighted that graphene restacking could lower epoxy SMP storage modulus to produce a low shape recovery force [38]. Thus, 2D graphene sheet fillers restrict the utilization of its excellent electrical and thermal properties to enhance SME in graphene-SMP composites. Three-dimensional (3D) graphene foam (GrF) as a filler is a potential solution to address the drawbacks of 2D graphene in polymer matrices. GrF is a 3D nano-carbon material with an interconnected continuous network of branches and nodes. Its framework permits GrF to be homogeneously distributed within a polymer matrix. Its macro-porous architecture allows GrF to have an ultralow density of <0.005 g/cm^3^, which is highly desirable for lightweight reinforcement. High surface energy and large specific surface area (850 m^2^/g) [42] of GrF can enable strong entanglements with epoxy SMP molecular chains. The physical cross-linking can strengthen the net points accountable for the permanent shape of epoxy SMP. Additionally, GrF’s high specific strength and elastic modulus can improve the stiffness of epoxy SMP [43]. This would consequently facilitate a step-up in the shape recovery force of GrF-based epoxy SMP composites.

Higher thermal conductivity (0.26–1.7 W/m-K) [44] and electrical conductivity (125 S/cm) [45] in GrF will provide fast phonon and electron pathways throughout the polymer due to the interconnected branch–node morphology. Hence, 3D graphene foam would improve epoxy SMP composites’ thermal conduction, electrical actuation, and recovery time. Despite the advantages of graphene foam as a nano-filler, only a few studies have investigated its potential to enhance the mechanical properties and shape memory effect (SME) of epoxy-based SMPs. Shivakumar et al. demonstrated that incorporating 0.1 vol.% of GrF into SMP epoxy increased thermal conductivity by 62%, thereby accelerating the SME [40]. Additionally, Rong et al. established the electro-activation of SME in SMPs through single-layer GrF [46].

Despite the progress in utilizing GrF to enhance the performance of epoxy-based SMPs, there remains a critical need for a comprehensive scientific understanding of the underlying mechanisms, particularly in the context of dual-triggering mechanisms involving heat and electricity. While adding GrF has demonstrated remarkable improvements in SMPs, such as increased thermal conductivity, enhanced mechanical strength, and superior shape memory effects, the specific interactions and structural changes responsible for these enhancements have not been fully elucidated. The primary objective of this study is to investigate the potential of GrF as an additive to shape memory epoxy, enabling the development of multifunctional structures, which can be thermally and electrically activated. To achieve this goal, epoxy SMP composites reinforced with varying weight percentages of GrF are fabricated using a mold-casting technique, forming different geometric shapes to exemplify their concept. To ensure the practical applicability of GrF-reinforced SMP composites as actuators, the samples underwent comprehensive analysis to assess the SME repeatability across multiple actuation cycles and temperatures, employing quantitative imaging techniques. The recovery rate behavior is also investigated near the glass transition temperature (T_g_) and correlated with thermal properties. Furthermore, the GrF-reinforced epoxy SMP composite is shaped into a bird-like structure, mimicking natural biological designs to investigate the crack opening and closure behavior during simulated wing flapping. Utilizing these advanced composites can revolutionize the design of smart materials and structures, introducing possibilities for innovative technologies and significantly improve performance across various industries.

## 2. Materials and Methods

### 2.1. Materials

Diglycidyl ether of bisphenol a epoxy monomer (DGEBA, EPON 826) (Mw = 340 g/mol) and neopentyl glycol diglycidyl ether (NGDE) (Mw = 216 g/mol) were obtained from Hexion Specialty Chemicals (Columbus, OH, USA) and Sigma-Aldrich (St. Louis, MO, USA), respectively. Curing agent poly(propylene glycol)bis(2-aminopropyl) ether (Jeffamine D230) (Mw = 230 g/mol) was purchased from Huntsman (Woodlands, TX, USA). The epoxy SMP components were used as received. Three-dimensional macro-porous, freestanding GrF with a pore size of 580 µm and 1–2 mm thickness was obtained from Graphene Supermarket (Calverton, NY, USA).

### 2.2. Fabrication of GrF-Epoxy SMP Composite by Mold Casting

EPON 826 was weighed into a ceramic cup and melted in an oven preset at 70 °C. This was followed by pouring the melted EPON 826 and a predetermined amount of Jeffamine D230 and NGDE into a cylindrical plastic container. The components were hand-mixed vigorously for 15 s. Freestanding GrF of 40 mm in length, 2 mm in width, and 1.2 mm in thickness was laid in an aluminum pan. The epoxy SMP mixture was poured into an aluminum pan, infiltrating the GrF. It is important to acknowledge that GrF is a delicate material, and cutting it to the desired shape and size presents significant challenges, as the foam can easily disintegrate under applied pressure. Therefore, the wt.%. of GrF varies depending on the shape and size of the samples required for specific tests. The GrF-epoxy SMP sample was cured at 100 °C for 1.5 h and post-cured at 130 °C for 1 h. Neat epoxy SMP samples were prepared similarly by casting as a control sample. After the curing process, the GrF-epoxy samples were demolded and prepared into different shapes for shape recovery testing. As the samples are cut into different shapes for various tests, the wt.% of GrF in the SMP matrix varies. However, the morphology of GrF remains the same throughout, which is the prime determinant for thermal and electrical conduction.

### 2.3. Microstructure and Phase Characterization

A JEOL JSM-6330F field emission scanning electron microscope (FESEM; Tokyo, Japan) was used to characterize the microstructure and interface of GrF-epoxy SMP composite. At an operating voltage of 20 kV and a working distance of 15 mm, the SEM imaged the macro-porous structure of GrF and the fracture surfaces of the composite specimen. Micro-Raman spectroscopy was conducted using a Spectra-Physics (noise and power drift stability of <1% and <3%, respectively; Model 3900S, CA, USA) equipped with Ti-sapphire crystal (514 nm) as the target. The Spectra-Physics also consists of a detector with 4:1 cm spectral resolution from Kaiser Optical Systems, Inc. (Ann Arbor, MI, USA), with a laser power of 18 mW, and a spot size of 2 μm. A micro-Raman spectroscopy study was conducted on composites to examine the graphene sheets’ interaction with SMP epoxy chain molecules. The FTIR spectra of SMP epoxy-based composites were recorded using a 4000-type spectrophotometer (JASCO FT/IR, 4100, Tokyo, Japan) from 4000 to 400 cm^−1^ to obtain the chemical signature.

### 2.4. Thermal Properties’ Characterization

SDT Q600 (accuracy ± 2%, TA instrument, Newcastle, Delaware, USA) was used to obtain a glass transition temperature of neat SMP epoxy and GrF-SMP composite. The specimens were heated to 200 °C at a heating rate of 100 °C/min in the argon environment. The T_g_ values of epoxy SMP and GrF-epoxy SMP composites were determined from the curves’ inflection temperatures. Thermal diffusivities and specific heat capacities of the epoxy SMP and GrF-epoxy SMP composites were measured by the flash method using NETZSCH LFA 467 HT HyperFlash (accuracy of thermal diffusivity ± 3%, accuracy of specific heat ± 3%, Burlington, MA, USA) within the temperature range of 25 to 70 °C. A circular sample of 11 mm diameter and 3 mm thickness was used for thermal diffusivity measurements. The GrF-epoxy SMP, when cut to the dimension mentioned above, had 0.13 wt.%. of GrF. Thermal conductivity was calculated by measuring the diffusivities, specific heats, and bulk densities of at least 5 samples.

### 2.5. Shape Memory Recovery Measurements

Shape memory recovery measurements were performed using (i) thermal and (ii) electrical actuation methods. Rectangular samples of epoxy SMP and GrF-epoxy SMP measuring 50 × 3 × 1 mm were placed on a hot plate at T_g_ + 28 °C for 5 s. The GrF-epoxy SMP had 0.75 wt.% of GrF. After removing it from the hot plate, the sample was manually deformed into a temporary ‘U’ shape with a radius of 3 mm in its rubbery state. Shape retention was allowed by promptly dipping the sample in a cold-water bath (20 °C) to lock the elastic deformation energy while maintaining the external constraint. The deformed, retained ‘U’ shape was ‘thermally’ actuated for shape recovery by placing it on the hot plate and using a hot air gun. The shape recovery was recorded using a digital camera. The images were extracted from the recorded video at different time intervals (e.g., 5, 15, 25, full recovery) and analyzed in terms of the change in the angle to quantify shape recovery.

For electrical stimulation, GrF was connected to a platinum wire (Pt) of 0.1 mm diameter (Surepure Chemetals, LLC, Florham Park, NJ, USA) using a conductive Pelco colloidal silver paste (Clovis, CA, USA). The silver paste’s addition between Pt wire and GrF allows contact resistance to be minimized during the current-induced heating of GrF. The silver paste was cured at 100 °C for 30 min. SMP epoxy was poured into GrF and cured as described in Section 2.2. The electrical stimulation was conducted on the rectangular shape of GrF-epoxy SMP composite using a four-probe technique employing a KEITHLEY Source Meter (accuracy 0.012%, Cleveland, OH, USA). The composite sample had 0.75 wt.% of GrF. Two pairs of probes, connected to the DC source for the current supply, were attached to the platinum wire electrodes in contact with the GrF-epoxy SMP composite. Raytek MX4+ infrared (IR) pyrometer (FLUKE, Santa Cruz, CA, USA) was employed to observe the surface temperature changes in response to the applied current. Step-wise currents (100–400 mA) were applied to the epoxy SMP composite to monitor its shape recovery response. The change in the shape was recorded through a digital video camera and analyzed for shape recovery. FLIR T620 High-Resolution Infrared Thermal Imaging camera (Wilsonville, OR, USA) was used to examine the change in the surface temperature and heat distribution through the embedded GrF in the composite. In both actuation techniques, at least 5 samples were tested, and the statistical significance of the data was established by the *t*-test method.

## 3. Results and Discussion

### 3.1. Microstructure of GrF-Shape Memory Epoxy Composite

**Figure 1:**
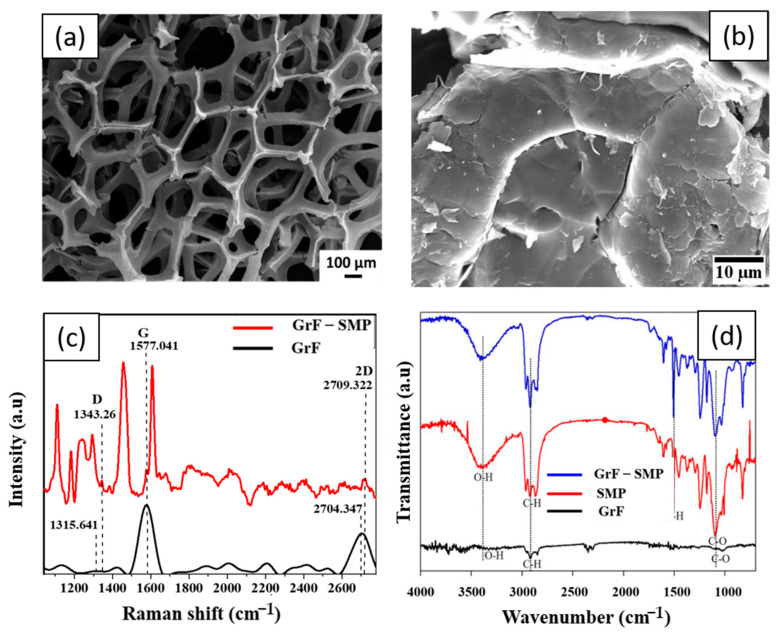
(**a**) Micrograph of as−received GrF, (**b**) Micrograph showing epoxy SMP infiltrated into the GrF exhibiting excellent interfacing (**c**,**d**) Micro-Raman spectroscopy and FTIR spectra of as-received GrF and GrF infiltrated with SMP, respectively.

Figure 1a shows the hierarchical structure of the as-received GrF, which reinforces epoxy SMP. GrF is a 3Dmacro-porous framework of interconnected graphene sheets synthesized via chemical vapor deposition. The fabrication of the composite requires liquid epoxy SMP infiltrating through the interconnected pores and wetting the branches and nodes of GrF. Figure 1b reveals that epoxy SMP percolates through the GrF’s pores and fills the entire hierarchical, macro-porous architecture. Most regions in Figure 1b also show epoxy SMP adhering to the GrF branches. In Supplementary Figure S1a,b, TEM images show a magnified view of the polymers’ excellent interfacing with the GrF. Curing epoxy SMP with a diamine cross-linker can influence the interface between the GrF and epoxy during and after polymerization. Effective addition reaction between the reactive groups of the epoxies and diamine allows for complete cross-linking. This is reflected in the interfacial appearance between the epoxy SMP and GrF, as shown in Figure 1b. The ability of epoxy SMP to wet and adhere to the interconnected graphene sheet network is necessary to facilitate easy phonon and electron transfer to improve the shape memory behavior for both thermal and electrical stimulation of the composites.

A micro-Raman spectroscopy study was conducted on GrF-epoxy composites to examine graphene sheets’ interaction with epoxy SMP chain molecules. Figure 1c shows the Raman measurement of pristine GrF, displaying three major characteristic signatures, including sp^2^ carbon–carbon bonds’ in-plane vibrations (G band), out-of-plane vibrations due to structural defects (D band), and second disorder band (2D band). The D peak’s significant low intensity compared with the G peak indicates that the graphene sheets are primarily defect-free [43,44,45,46,47,48,49,50,51,52]. The D and 2D peaks of pristine GrF are found at 1315.64 and 2704.35 cm^−1^, respectively. However, impregnation of GrF with epoxy SMP shifted the D and 2D peaks to the right with an assigned value of 1343.24 and 2709.32 cm^−1^, respectively. This implies the physical interaction of the backbone chain of epoxy SMP with a network of graphene sheets. Such interaction may result in bond length adjustment of the epoxy SMP molecules.

Figure 1d shows the FTIR spectra of epoxy SMP, GrF, and GrF-epoxy SMP composite. Absorption peaks in the epoxy SMP spectrum are revealed at 3348, 2970, 1535, and 1100 cm^−1^, attributed to stretching and bending vibrations from O-H, C-H, N-H, and C-O, respectively [53]. The GrF spectrum shows characteristic peaks of some functionalities, such as C-O at 1100 cm^−1^, C-H at 2970 cm^−1^, and O-H at 3348 cm^−1^ [54]. The GrF-epoxy SMP absorption band shows that epoxy SMP peaks are distinct, overshadowing the weak peaks of GrF. From the FTIR spectra, it can be seen that interfacial intermolecular interactions occur between the functionalities on GrF and epoxy SMP. The O-H group can easily be attached to graphene edges and initiate electrostatic attractions with O-H and N-H groups from epoxy SMP. This creates hydrogen bonding between the graphene and epoxy SMP. Hydrogen bonding interactions between hydroxyls and hydroxyl-amide groups result in the formation of hydrogen bond strengths. The typical intermolecular hydrogen bond strength between hydroxyl and amide groups is 29 kJ/mol, and between two hydroxyl groups, it is 21 kJ/mol [55]. Thus, strong hydrogen bond attractions between these functionalities could account for the bond length adjustment.

### 3.2. Glass Transition of GrF-Epoxy SMP Composites

**Figure 2:**
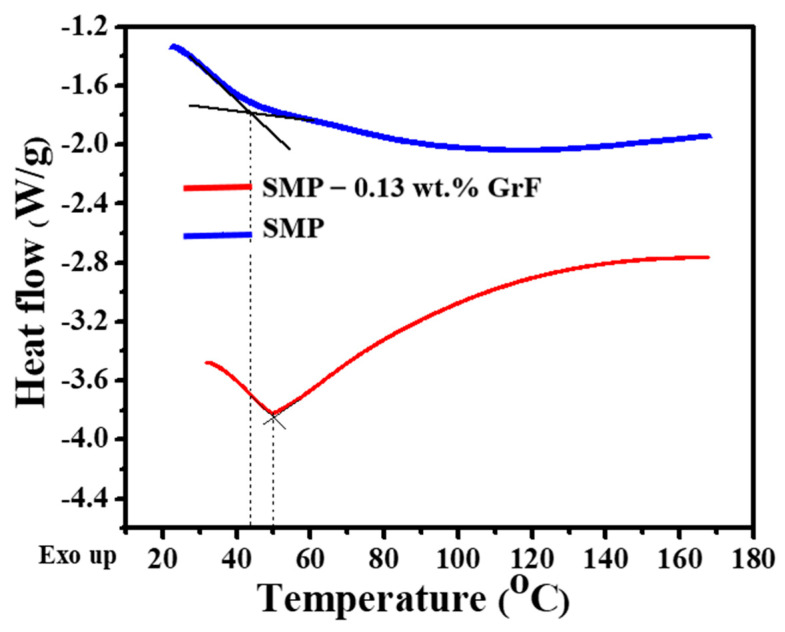
Differential scanning calorimetry of SMP epoxy and SMP epoxy−0.13 wt.% GrF composite displaying their glass transition behaviors.

Figure 2 illustrates the thermal transition characteristics of epoxy SMP and GrF-epoxy SMP composites. The T_g_ of the epoxy SMP is measured at 42 °C. Adding GrF filler to the epoxy SMP composite, specifically at 0.13 wt.%, increases the T_g_ to 50 °C, indicating a 19% improvement. GrF has a high aspect ratio and large surface area, providing numerous opportunities for strong interfacial interactions with the epoxy matrix. The functional groups on the surface of GrF can form hydrogen bonds with the epoxy resin, as shown in Figure 1, leading to improved interfacial adhesion. These enhanced interactions contribute to the restriction of molecular chain mobility, increasing T_g_. Moreover, the 3D interconnected framework provided by GrF within the epoxy SMP matrix promotes the entanglement of terminal hydroxyl groups in the side chains of epoxy SMP. This entanglement immobilizes the surrounding chains, preventing cross-linking and leading to a higher T_g_. The enhanced physical interaction between the epoxy SMP’s terminal groups and the 3D graphene sheets network causes the epoxy SMP chain to wrap around the branches and nodes of GrF. Consequently, moving confined epoxy SMP chains from a glassy to a rubbery state requires more thermal energy. The increased T_g_ is advantageous, as it prevents shape recovery degradation of epoxy SMP after thermal cycling and expands the temperature range for structural aerospace applications.

Additionally, the addition of 0.13 wt.% GrF into SMP epoxy significantly reduces the width of the T_g_ transition, indicating restricted chain conformations and reduced relaxation time distributions. This narrower transition temperature in the GrF-epoxy SMP composite promotes faster shape recovery rates. In comparison, a broader transition temperature in SMP epoxy can lead to incomplete shape fixing and slower recovery.

### 3.3. Thermal Actuation of GrF-Epoxy SMP Composites

**Figure 3:**
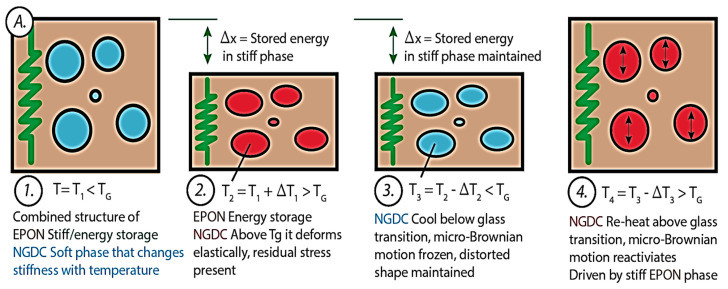

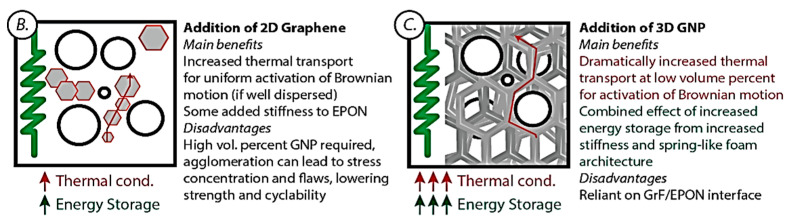
(**A**) Illustration of the dual-component mechanism (DCM) in SMP epoxy, (**B**) Dual-component mechanisms with enhanced performance for 2D graphene addition, (**C**) The mechanisms of improved performance for 3D graphene-foam-based additions.

The shape recovery of epoxy SMP and GrF-epoxy SMP composites is evaluated through direct heating. Direct heating with a hot plate is a widely recognized method for thermally activating SMP epoxy, while a heat gun can also be used. The shape memory behavior of SMP epoxy under direct heating is governed by its dual-component mechanism. Figure 3A illustrates the molecular-level shape memory effect of SMP epoxy. Upon heating from room temperature (T1) to a temperature above the triggering temperature (T2), stress is applied (T2 > T_g_), resulting in the elastic deformation of NGDE chains and energy storage within the EPON stiff phase.

The SMP epoxy is cooled below T_g_ (T3 < T_g_), and the stress is removed, causing the NGDE polymer segments to cool and freeze. This freezing allows the SMP epoxy to retain its temporary shape. The shape memory effect (SME) is triggered when T3 > T_g_, where reheating reactivates the frozen micro-Brownian motion, fully recovering the distorted polymer molecules. Figure 3B illustrates that the addition of 2D graphene nanoplatelets forms a dense network within the epoxy matrix, improving the mechanical properties and thermal conductivity thus enabling faster and uniform shape recovery. Strong interfacial interactions between graphene nanoplatelets and the epoxy matrix enhance stability and durability. Similarly, Figure 3C shows that the incorporation of 3D GrF increases the volume of the epoxy matrix, enhancing energy storage capacity, shape recovery, and mechanical properties. The interconnected graphene network facilitates efficient heat transfer and improves shape fixity. The evaluation of the SMP epoxys’ performance considers shape recovery behavior with respect to temporal and thermal gradients.

#### 3.3.1. Temporal Gradient Behavior of SMP Epoxy-GrF Composite

**Figure 4:**
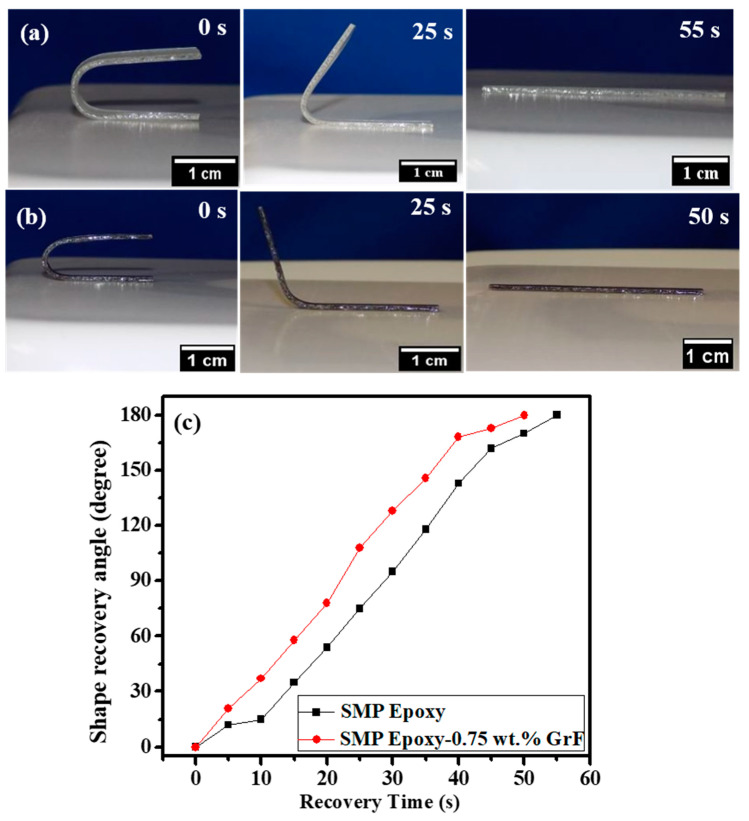
Shape recovery images of (**a**) SMP epoxy and (**b**) SMP epoxy-0.75 wt.% GrF composite triggered by direct heating from the hot plate. (**c**) Shape recovery angle as a function of time for SMP epoxy and SMP epoxy-0.75 wt.% GrF composite subjected to direct heating from the hot plate.

The SMP epoxy samples’ temporal gradient is qualitatively assessed by subjecting them to direct heating via the hot plate. Figure 4a,b present the fold-deploy shape recovery test of SMP epoxy and GrF-epoxy SMP epoxy composite with 0.75 wt.% GrF, respectively, at 70 °C.

The SMP epoxy composite exhibits a significantly higher temporal gradient than SMP epoxy. The temporal gradient of the SMP epoxy samples is quantified in terms of their recovery angle, as determined by the shape recovery ratio equation (Equation (1)). The recovery angle can be expressed as follows:

(1) $$\mathrm{Shape}\;\mathrm{recovery}\;\mathrm{ratio}:\;R_{r}=\frac{\theta_{deformation}-\theta_{residual}}{\theta_{deformation}}\times 100$$

$$\theta_{residual}=\mathrm{residual angle during recovery state}$$

$$\theta_{deformation}=\mathrm{angle of bending deformation}$$

Figure 4c, which depicts the shape recovery ratio, demonstrates the temporal gradient of a fold-deploy test for GrF-epoxy SMP composites. The plot clearly illustrates that SMP epoxy and its composite display complete shape recovery behavior. By analyzing the recovery angle over time, the temporal gradients of the samples can be assessed (Figure 4c). The results indicate that the GrF-epoxy SMP composite exhibits a higher temporal gradient (recovery rate of 4.93°/s) compared to SMP epoxy (4°/s), indicating a 23% faster recovery due to the seamless phonon transfer facilitated by the branch–node framework of GrF. Moreover, the high aspect ratio and large surface area of GrF facilitate strong interfacial interactions with the epoxy matrix. The resulting improved interfacial adhesion enhances the transfer of stress and strain between the GrF and epoxy phases. This efficient stress transfer mechanism can facilitate faster shape recovery and contribute to the observed higher recovery rate.

**Figure 5:**
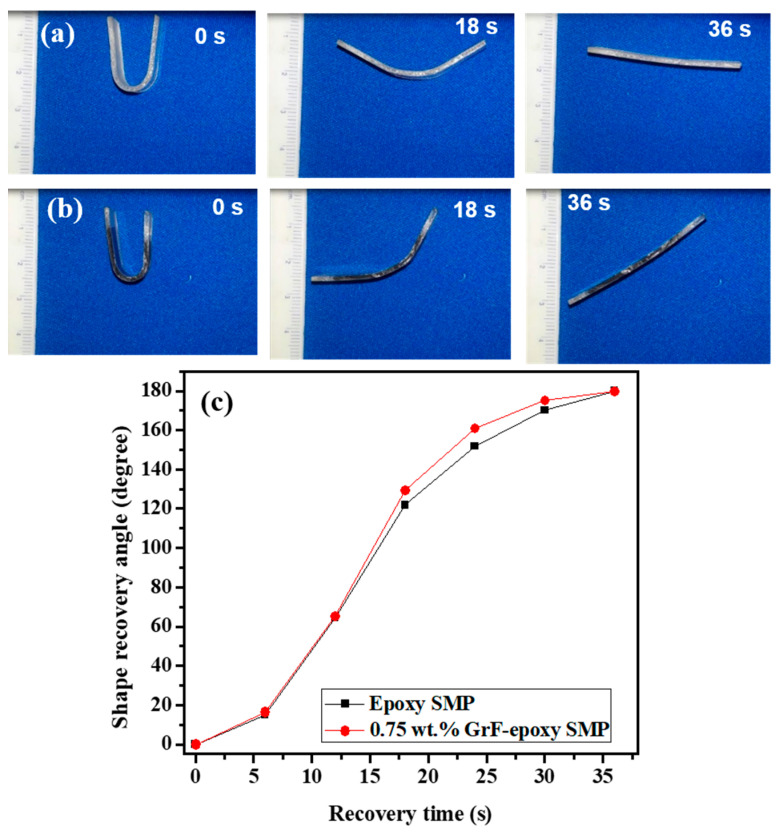
Shape recovery demonstration of thermally actuated (heated air) (**a**) SMP epoxy and (**b**) SMP epoxy-0.75 wt.% GrF composite stimulated by heated air from the heat gun. (**c**) Shape recovery angle as a function of time for SMP epoxy and SMP epoxy-0.75 wt.% GrF composite exposed to hot air from the heat gun.

Figure 5a,b demonstrate the recovery images of bent shapes for epoxy SMP and its composite when stimulated by hot air from a heat gun at a temperature of 70 °C. Both epoxy SMP and its composite regain their original shapes within a recovery time of 36 s when stimulated above their T_g_. Based on Figure 5c, epoxy SMP and its composite exhibit average recovery rates of 7.6°/s and 8.0°/s, respectively. When SME is thermally actuated using uniformly distributed hot air, the SMP composite exhibits a 40% increase in the recovery rate. GrF’s exceptional thermal conductivity, coupled with the uniform heating of the sample, allows instantaneous and more efficient heat transfer throughout the bulk of the composite, ensuring that the entire material reaches the required temperature for shape recovery more quickly. This indicates that the GrF-epoxy SMP composite exhibits superior and faster shape recovery characteristics when employing different direct heating approaches than epoxy SMP.

The EPON phase of epoxy SMP plays a crucial role in its shape recovery, providing the necessary energy for the material to return to its permanent shape. The covalent interactions between the amine and epoxide groups in the EPON segment contribute to epoxy SMP’s high shape recovery rate of 97.35%. However, adding GrF to epoxy SMP enhances the shape recovery performance, achieving 100% shape recovery in the composite. The GrF framework, composed of high-modulus graphene sheets, adds stiffness to the net points within the epoxy SMP. The uniform dispersion of GrF throughout the SMP matrix enables the distribution of stiffness, thanks to strong interfacial interactions between the graphene sheets and epoxy SMP. The attractive van der Waals forces between the graphene layers also contribute to the energy storage capacity of the composite, allowing for maximum shape recovery. The combined influence of the EPON net points and the stiffness and energy storage of GrF enables 100% shape recovery in the GrF-epoxy SMP composite.

#### 3.3.2. Effect of Switching Temperatures on the Shape Recovery Rate

Switching temperatures for shape recovery refers to T_g_ and temperatures around the T_g_ region. They are an essential parameter in the thermal actuation of epoxy SMP and GrF-epoxy SMP composite.

**Figure 6:**
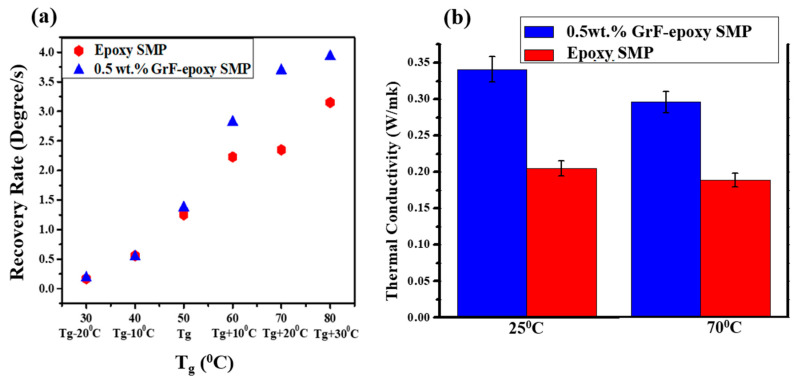
(**a**) Shape recovery rate of SMP epoxy and SMP epoxy-0.5 wt.% GrF as a function of switching temperatures. (**b**) Thermal conductivity behavior of SMP epoxy and SMP epoxy-0.5 wt.% GrF at 25 and 70 °C.

Figure 6 illustrates the shape recovery rate evolution of epoxy SMP and GrF-epoxy SMP at different switching temperatures. GrF-epoxy SMP consistently exhibits a faster recovery rate than epoxy SMP across all switching temperatures. This outcome highlights the enhanced heat conduction within the GrF-epoxy SMP composite facilitated by the efficient phonon transfer across the interconnected node–branch network. The improved heat conduction within the graphene foam enables the unlocking of NGDE chain molecules, releasing stored energy in the GrF and EPON. The comparison of recovery rates in Figure 4c and Figure 5c indicate that the highest recovery rate is achieved at the switching temperature of T_g_ + 20 °C. This suggests that supplying thermal energy at T_g_ + 20 °C ensures sufficient heat distribution to initiate shape recovery in both epoxy SMP and GrF-epoxy SMP composite, making T_g_ + 20°C the optimal triggering temperature for shape recovery.

Figure 6b displays the thermal conductivities of epoxy SMP and GrF-epoxy SMP composites measured using the flash diffusivity method. The composite SMP has 0.5 wt.% of GrF. The thermal conductivity of epoxy SMP is 0.189 W/m-K, while the GrF-epoxy SMP composite exhibits a higher thermal conductivity of 0.296 W/m-K, representing a 57% increase. This higher thermal conductivity of the GrF-epoxy SMP composite contributes to its 23% faster recovery rate than epoxy SMP (Figure 4). The incorporation of GrF within the epoxy SMP composite results in a notable enhancement of its shape recovery rate. This improvement can be attributed to the superior heat conduction facilitated by the interconnected node–branch network of the GrF. GrF’s inherent structure allows for efficient thermal energy transfer, enabling effective unlocking of the non-covalent dynamic entanglement (NGDE) chain molecules in the GrF and epoxy resin. Consequently, the stored energy within the GrF and epoxy resin is more rapidly released, promoting a faster shape recovery process than the pure epoxy SMP. This observation underscores the pivotal role played by enhanced thermal conductivity in achieving superior shape recovery performance in the GrF-epoxy SMP composite.

#### 3.3.3. Influence of Thermo-Mechanical (T-M) Cycles on the Glass Transition Temperature

**Figure 7:**
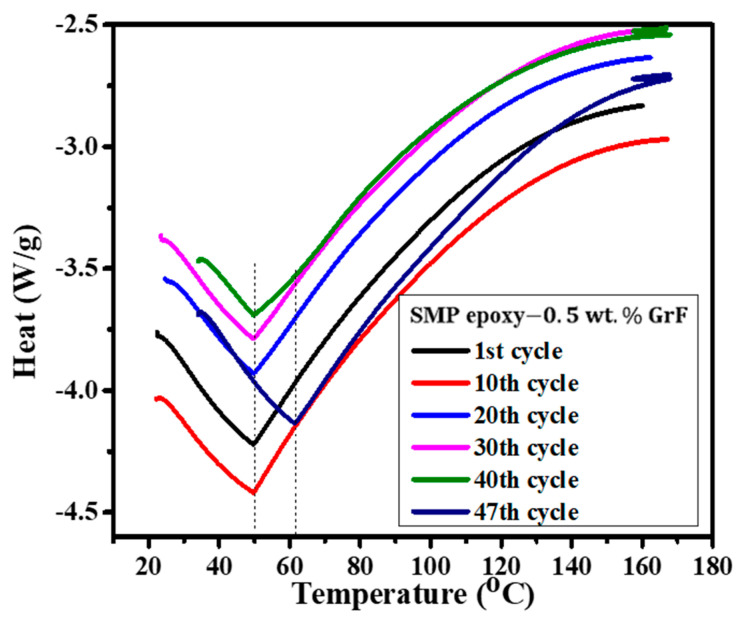
Glass transition behavior of SMP epoxy−0.5 wt.% GrF as a function of T-M cycles.

Figure 7 demonstrates the ability of GrF integration to increase the T_g_ of epoxy SMP composite. Additionally, the T-M cycling process influences the T_g_ behavior of the GrF-epoxy SMP composite, as revealed in Figure 7. The first 30 applied T-M cycles do not affect the T_g_. However, the 47th T-M cycle exhibits a significant increase in T_g_ from 50 °C to 58 °C, corresponding to a 16% T_g_ increase in the graphene-foam-epoxy SMP composite. The constant T_g_ observed during the initial T-M cycles may be attributed to the constrained entanglement of GrF with the side chains of epoxy SMP. As the number of T-M cycles increases, the physical interaction between GrF and the backbone chain of epoxy SMP intensifies, resulting in the confinement of the backbone chain’s internal rotation. This confinement leads to a 16% T_g_ increase in the GrF-epoxy SMP composite during the 47th T-M cycle. Consequently, T-M cycling provides a means to tailor the T_g_ of the graphene-foam-epoxy SMP composite.

### 3.4. Electrical Actuation of Epoxy SMP and GrF-Epoxy SMP Composite

A direct thermal source for the actuation of shape change in epoxy SMP limits its practical applications. The induction of heat through electricity (indirect heating) as an alternative trigger method in epoxy SMP is essential. Graphene as a carbon-based filler has been considered to trigger epoxy SMP electrically [36]. However, graphene’s high electrical conductivity can be compromised because of its high inter-sheet contact resistance and restacking issues. Hence, 3D GrF filler is considered to electrically stimulate epoxy SMP for improved shape memory behavior of the epoxy SMP composite. To demonstrate that epoxy SMP is a virtually non-electrically conductive polymer, epoxy SMP is subjected to Joule heating. Infrared thermal imaging is used to observe induced heating on epoxy SMP. A current of 200 and 400 mA is applied for 25 s.

**Figure 8:**
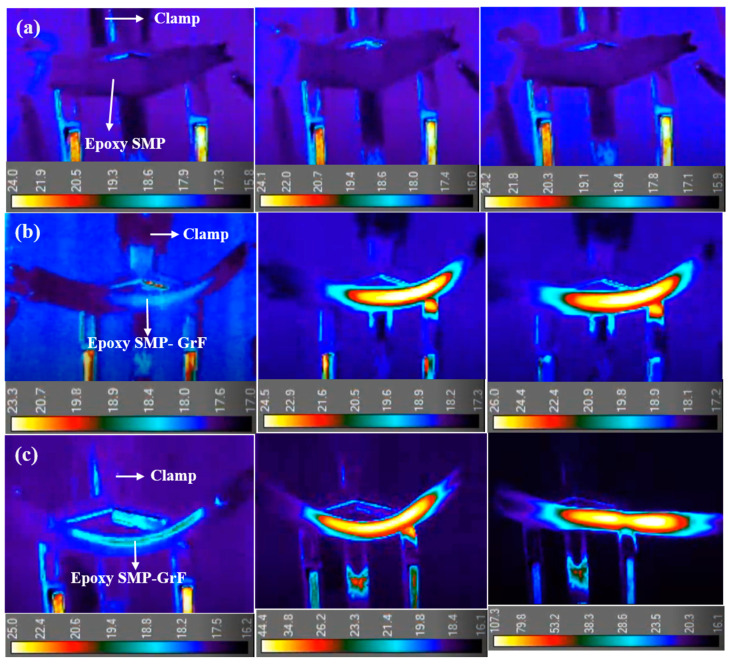
(**a**) Images of temperature distribution through epoxy SMP at 70 °C under applied current of 400 mA, (**b**,**c**) Infrared images revealing electro-thermal heating of composite resulting in heat flow through embedded graphene foam in SMP epoxy matrix under applied current of 200 and 400 mA, respectively.

Figure 8a shows the infrared thermal images of epoxy SMP. The consistent surface temperature indicates no heat generation, suggesting a lack of heat conduction in the material. Therefore, the epoxy SMP cannot self-electrically stimulate or exhibit shape memory behavior. However, when GrF is added to the epoxy SMP, it acts as a pathway for the current and enables electrical activation. When currents of 200 and 400 mA are passed through the GrF embedded in the epoxy SMP, as depicted in Supplementary Figure S2, the GrF acts as a resistive element, generating heat, which activates the molecular segments of the epoxy SMP matrix. Heat induces motion in the molecules and triggers the recovery of the epoxy SMP from its temporary shape to its original shape. Figure 8b,c show infrared thermal images of the electrical stimulation of the GrF-epoxy SMP composite. The heat distribution through the GrF gradually increases as the applied current rises. The branch–node structure of the GrF facilitates heat transport through the composite. The strong interface between the GrF and epoxy SMP enhances thermal conductance, enabling efficient heat transfer from the GrF to the epoxy SMP.

Increasing the applied current from 200 mA to 400 mA intensifies the heat radiation through the GrF, emphasizing its role as an efficient resistive heating element in the epoxy SMP. This demonstrates that GrF effectively conducts current-induced heat into the epoxy SMP, making it a suitable conductive filler for triggering shape memory behavior. A 90% shape recovery is achieved with a maximum applied current of 400 mA, which can be attributed to the interfacial thermal resistance (ITR) between the GrF and epoxy SMP. A simple thermal equation (Equation (2)) can be used to determine the ITR between GrF and epoxy SMP, as shown below [56]:

(2) $$R=\frac{∆TA}{Q}$$

Determining parameter R, which represents the resistance at the interface, plays a crucial role in controlling the heat transport across the GrF–epoxy SMP interface. This determination is influenced by factors such as the temperature drop (∆T) across the interface, the cross-sectional area (A), and the total heat transfer (Q) across the interface. The ITR can lead to phonon vibrational mismatch and scattering at the GrF–epoxy SMP interface during phonon transfer. This implies that the nano-scale contact area between the GrF and epoxy SMP is responsible for the shape recovery observed in electrically actuated GrF-epoxy SMP composites.

### 3.5. Self-Healing Effect in Bird’s Wing Made of GrF-Epoxy SMP Composite

Epoxy SMP and its composites possess remarkable structural versatility, making them highly sought-after smart polymers in engineering applications. For instance, epoxy SMP and GrF-epoxy SMP composites were engineered into S, L, inverted U, and accordion shapes. By utilizing hot water or hot air, these shapes could be temporarily deformed and triggered back to their original shape (see Supplementary Figures S3–S5). The accordion shapes made with GrF-epoxy SMP exhibited faster recoverability than pure epoxy SMP due to the superior thermal and mechanical properties of GrF (refer to Supplementary Video S1). Thus, GrF-epoxy SMP composites hold great potential in applications such as actuators, piezoelectric devices, deployable reflectors, morphing wings, and masts due to their high shape deformability. Inspired by the wings of birds, we successfully fabricated stimulus-responsive birds using epoxy SMP and GrF-epoxy SMP composites.

**Figure 9:**
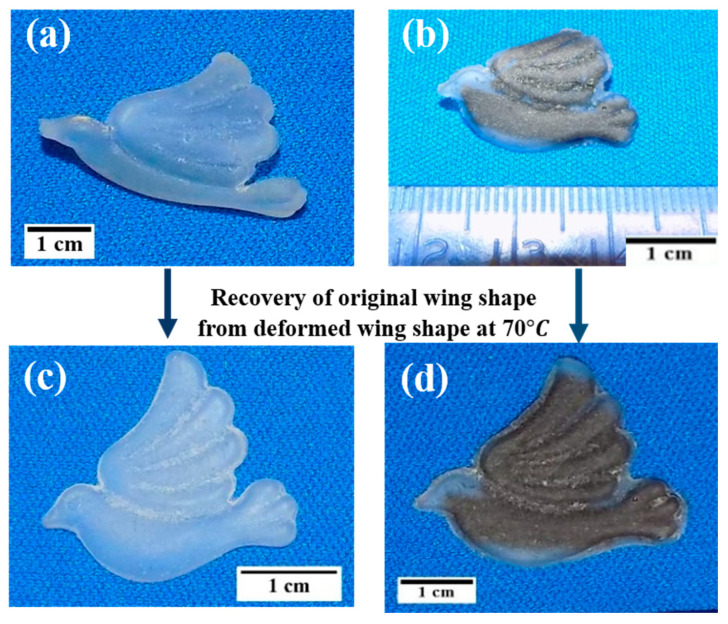
(**a**,**b**) Images of deformed (temporary shape) wings of epoxy SMP bird and GrF-epoxy SMP bird at 25 °C, respectively. (**c**,**d**) Images showing recovered wings of epoxy SMP bird and GrF-epoxy SMP bird at 70 °C, respectively.

Figure 9a,b depict pre-deformed epoxy SMP and GrF-epoxy SMP composite wings, respectively. These wings exhibit high flexibility during deformation above their T_g_ of 70 °C. When cooled to room temperature, the wings retain their deformed shape. To trigger the recovery of the wings to their original shape, hot water at 70 °C is used as a stimulus. Figure 9c,d demonstrate the recovery of the epoxy SMP and GrF-epoxy bird wings, respectively. The GrF-epoxy SMP bird wing recovers faster than the pure epoxy SMP bird wing due to the additional energy storage the GrF provides (see Supplementary Video S2).

**Figure 10:**
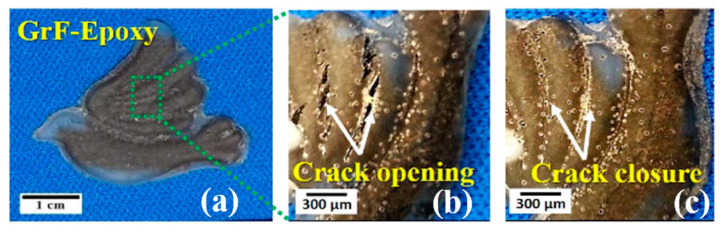
(**a**,**b**) Image of a cracked-open wing of biomimetic graphene-foam-epoxy SMP bird after 15th T-M cyclic deformation. (**c**) Images of the crack-closed wing of graphene-foam-epoxy SMP bird.

Figure 10a,b reveal surface cracks after multiple thermal-mechanical cyclic deformations of the GrF-epoxy composite bird wing. However, it is important to note that the major cracks close during the shape recovery process under heat stimulation, as shown in Figure 10c (see Supplementary Video S3). This behavior can be attributed to multiple structural and dynamic factors within the GrF-epoxy SMP composite. Despite the formation of surface cracks, the integrity of the EPON phase net points in the epoxy SMP bird wing remains intact. The GrF filler acts as a crack-bridging agent, preventing the disintegration of the net points and transferring energy to the crack surface through its hierarchical structure. This prevents failure of the GrF-based epoxy SMP bird wing.

Furthermore, heat-induced dynamic factors cause adjustments in the chain conformations of the EPON phase around the crack region of the GrF-epoxy SMP bird wing. These conformation adjustments unlock energy stored within the EPON phase, contributing to recovering the wing’s original shape. Additionally, the release of stored energy within the GrF aids in closing the cracks in the GrF-epoxy SMP bird wing. The concept demonstrated by the GrF-epoxy SMP bird highlights the potential of GrF-epoxy SMP composites in designing morphable aircraft wings. These composites can serve as multifunctional hierarchical materials in aircraft components, particularly for morphing wings [56] and deicing applications [57]. These promising applications underscore the capability of graphene foam reinforcement in epoxy SMP to create robust, lightweight, and multifunctional advanced polymer nanocomposites.

## 4. Conclusions

The findings of this study contribute to addressing the limitations of slow recovery due to inferior thermal properties associated with SMPs. The incorporation of GrF within the epoxy SMP matrix facilitated enhanced heat conduction through its interconnected 3D network, leading to a significant 57% increase in thermal conductivity. As a result, the GrF-epoxy SMP composite exhibited a 23% faster recovery rate than the pure SMP epoxy. The GrF-epoxy SMP composites exhibited enhanced shape recovery behavior, with shape recovery of ~97% for thermally triggered samples. The study also established that as the number of thermo-mechanical cycles increases, the physical interaction between GrF and the backbone chain of epoxy SMP intensifies, resulting in a 16% increase in the T_g._ Furthermore, the GrF-epoxy SMP composite exhibited remarkable electrical conductivity, enabling dual-triggering capabilities. A 90% shape recovery is achieved with a maximum applied current of 400 mA due to the interfacial thermal resistance (ITR) between the GrF and epoxy SMP, visualized by thermal imaging. The design and programming of biomimetic bird-shaped composites demonstrated a faster recovery time and crack opening and closure behavior after multiple thermo-mechanical cycles. This behavior highlights the potential of the GrF-epoxy SMP composite as a self-healing material. The GrF-based SMP epoxy composites presented in this research offers promising opportunities for various applications, including mechanical systems, wearable sensors, morphing wings, foldable robots, and antennas. These composites, responsive to stimuli and featuring enhanced properties, have the potential to revolutionize the field of smart polymeric materials and inspire further advancements in the design of functional structures.

## 5. Patents

Agarwal, A., Thomas, T., Idowu, A., and Boesl, B., Florida International University FIU, 2021. Shape memory polymer inks and methods of printing the same. U.S. Patent 10,906,238.

## Data Availability

Please contact the corresponding author to request the data presented in this study.

## Supplementary Materials

The following supporting information can be downloaded at: https://www.mdpi.com/article/10.3390/polym15132903/s1, Figure S1: (a) TEM image showing graphene wrinkles at the interface of GrF and SMP. (b) TEM image showing excellent interfacing between the GrF and SMP. Inset shows SEM image (Figure 1b) presented in the manuscript; Figure S2: Schematic showing electrical actuation showing pre-deformed GrF-epoxy SMP composite; Figure S3: (a) Images of epoxy SMP fabricated to S-shape, deformed to thin rec-tangular shape and recovered back to S-shape under the influence of external heat stimulus; (b) Images of epoxy SMP designed to L-shape, deformed into rectangular shape and recovered back to L-shape stimulated by heat; (c) Images of GrF-epoxy SMP made into inverted U-shape deformed into irregular rectangular shape and recovered back to inverted U-shape when stimulated by heat; Figure S4: (a) Images of epoxy SMP fabricated into accordion-like shape, stretched out into temporary shape and restored to its original shape under the influence external heat from hot water; (b) Images of graphene nano platelet -epoxy SMP made into accordion-like shape, deformed into temporary shape and recovered to its original shape when triggered by thermal actuation; (c) Images of GrF-epoxy SMP composite made into accordion-like shape, deformed into temporary shape and recovered to its original shape when stimulated under heat; Figure S5: (a) Images of epoxy SMP fabricated into accordion-like shape, stretched out into temporary shape and restored to its original shape under the influence of hot air; (b) Images of GrF-epoxy SMP made into accordion-like shape, deformed into temporary shape and recovered to its original shape when triggered by hot air.
