# Supplementary material for: Electrically and Thermally Triggered Three-Dimensional Graphene-Foam-Reinforced Shape Memory Epoxy Composites
Source: Polymers (Basel). 2023 Jun 30;15(13):2903. doi: 10.3390/polym15132903 (PMC10346916; doi:10.3390/polym15132903)
Supplement: Supplementary file 1 [file polymers-15-02903-s001.zip › polymers-2452915-supplementary.pdf]

Supporting Information

Electrically and Thermally Triggered Three-Dimensional Graphene Foam-Reinforced Shape Memory Epoxy Composites

Adeyinka Idowu, Tony Thomas, Jenniffer Bustillos, Benjamin Boesl, and Arvind Agarwal*

Plasma Forming Laboratory, Department of Mechanical and Materials Engineering, Florida International University, Miami, Florida 33174, United States

*Corresponding Author: Dr. Arvind Agarwal; agarwala@fiu.edu

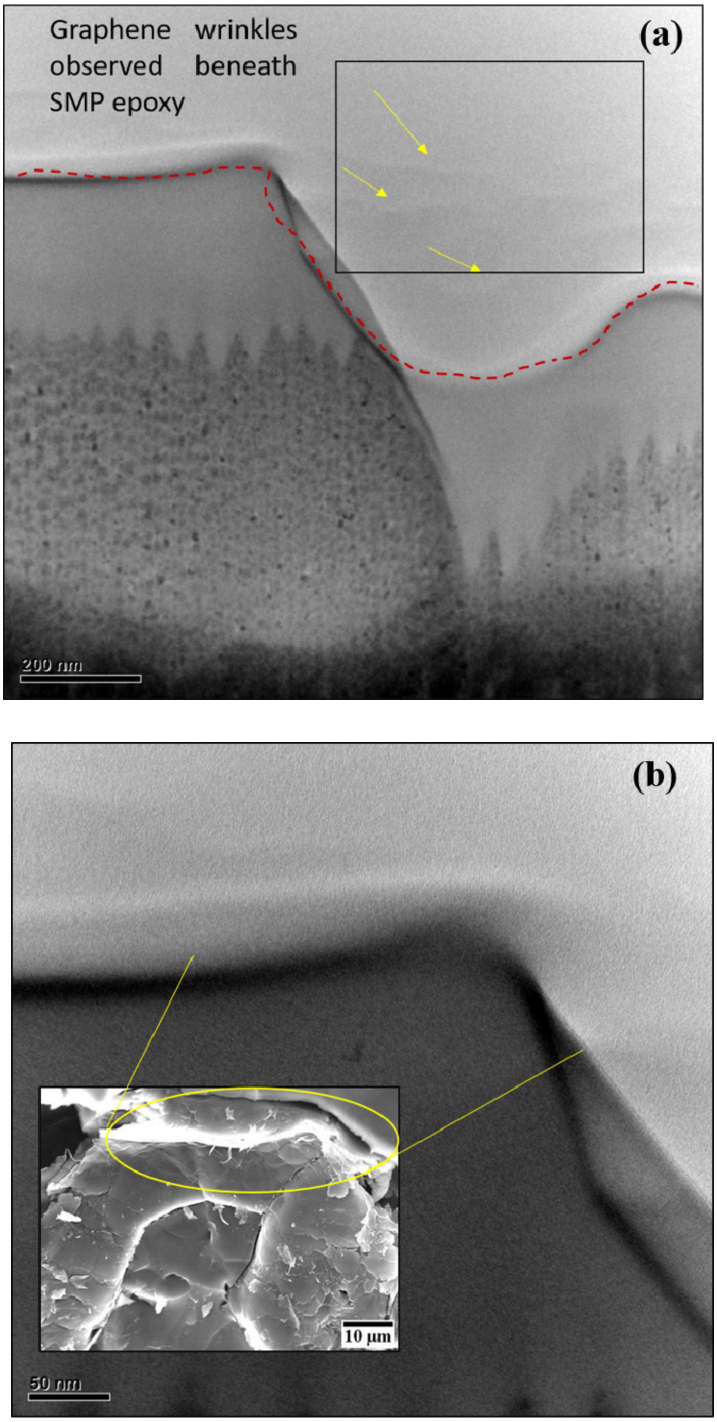

Figure S1. (a) TEM image showing graphene wrinkles at the interface of GrF and SMP. (b) TEM image showing excellent interfacing between the GrF and SMP. Inset shows SEM image (Figure 1b) presented in the manuscript

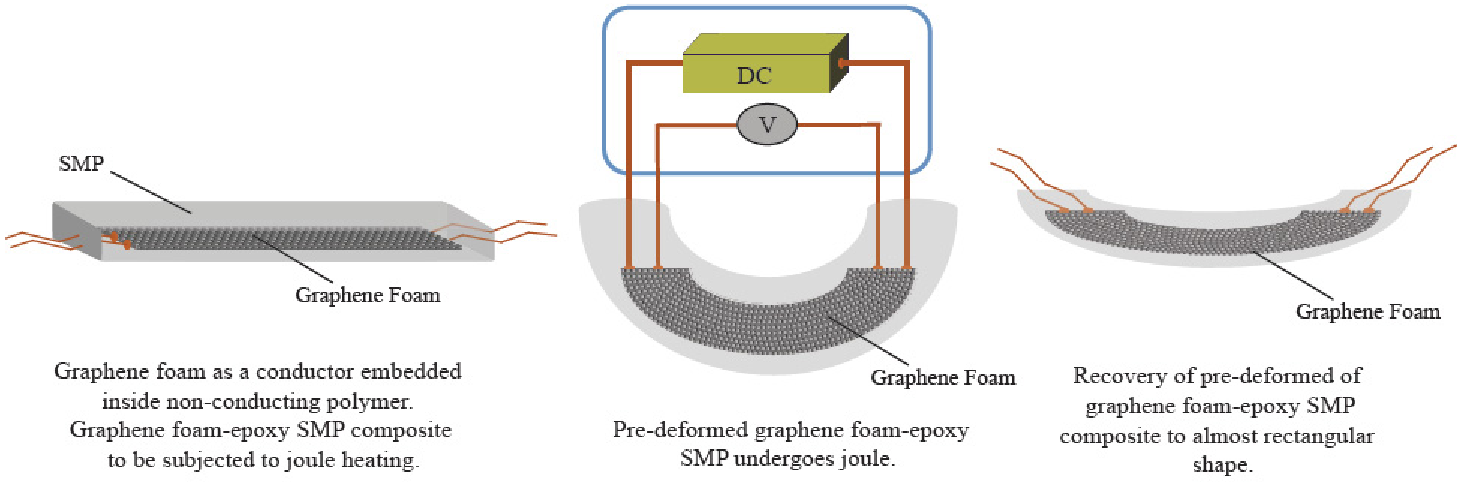

Figure S2. Schematic showing electrical actuation showing pre-deformed GrF-epoxy SMP composite

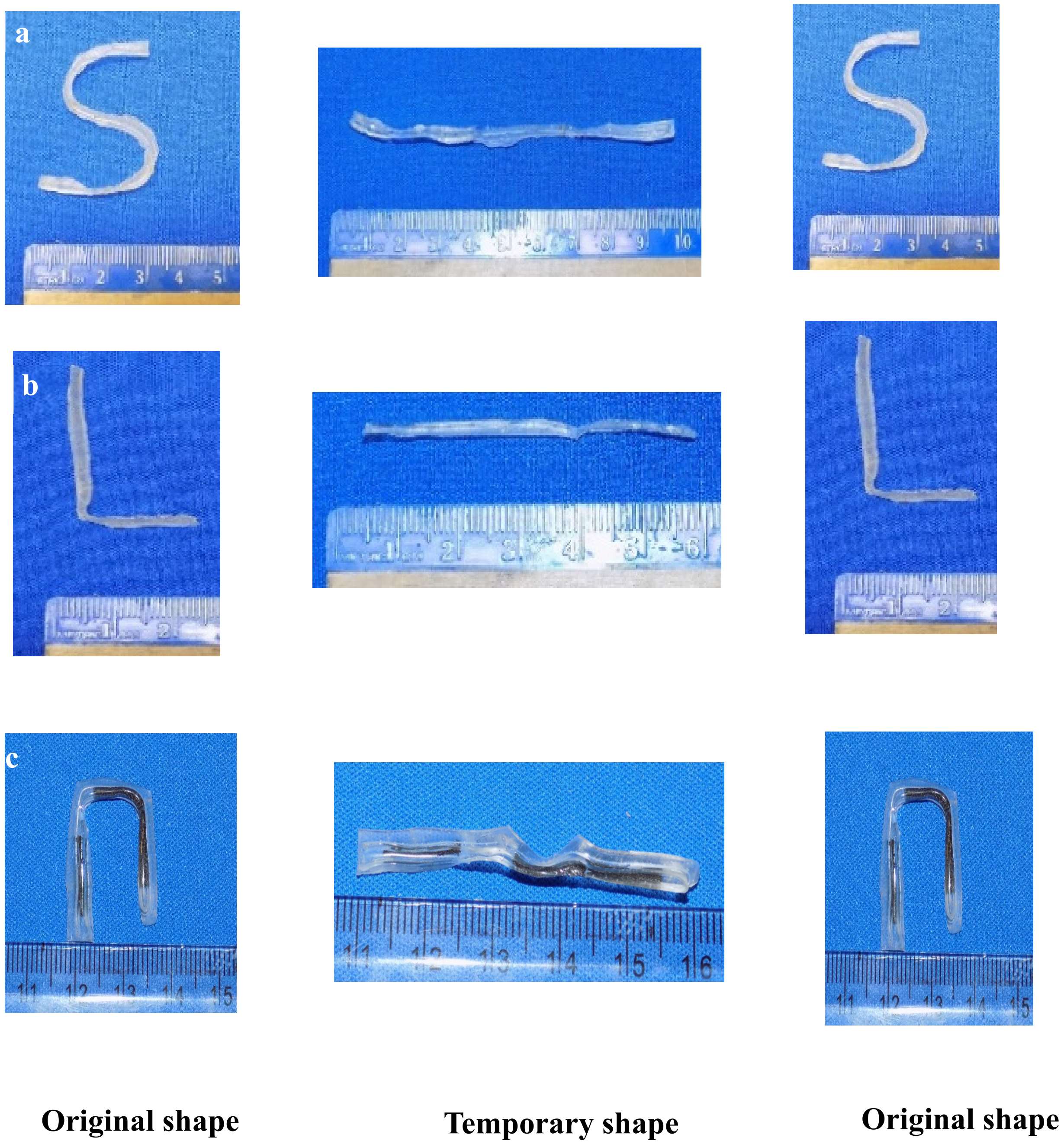

Figure S3. a) Images of epoxy SMP fabricated to S-shape, deformed to thin rectangular shape and recovered back to S-shape under the influence of external heat stimulus; b) Images of epoxy SMP designed to L-shape, deformed into rectangular shape and recovered back to L-shape stimulated by heat; c) Images of GrF-epoxy SMP made into inverted U-shape deformed into irregular rectangular shape and recovered back to inverted U-shape when stimulated by heat

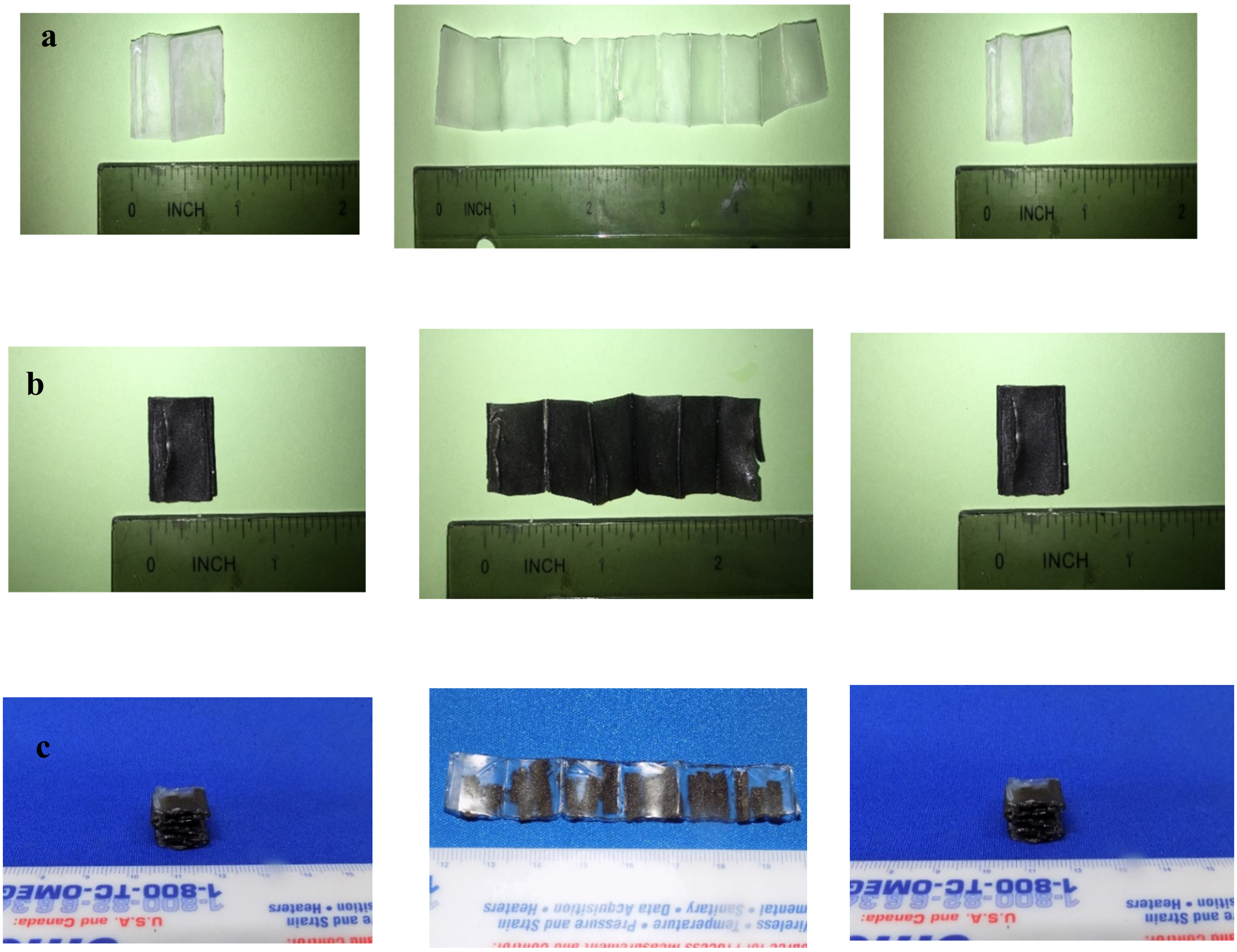

Original shape **Temporary shape** **Original shape**

Figure S4. a) Images of epoxy SMP fabricated into accordion-like shape, stretched out into temporary shape and restored to its original shape under the influence external heat from hot water; b) Images of graphene nano platelet -epoxy SMP made into accordion-like shape, deformed into temporary shape and recovered to its original shape when triggered by thermal actuation; c) Images of GrF-epoxy SMP composite made into accordion-like shape, deformed into temporary shape and recovered to its original shape when stimulated under heat

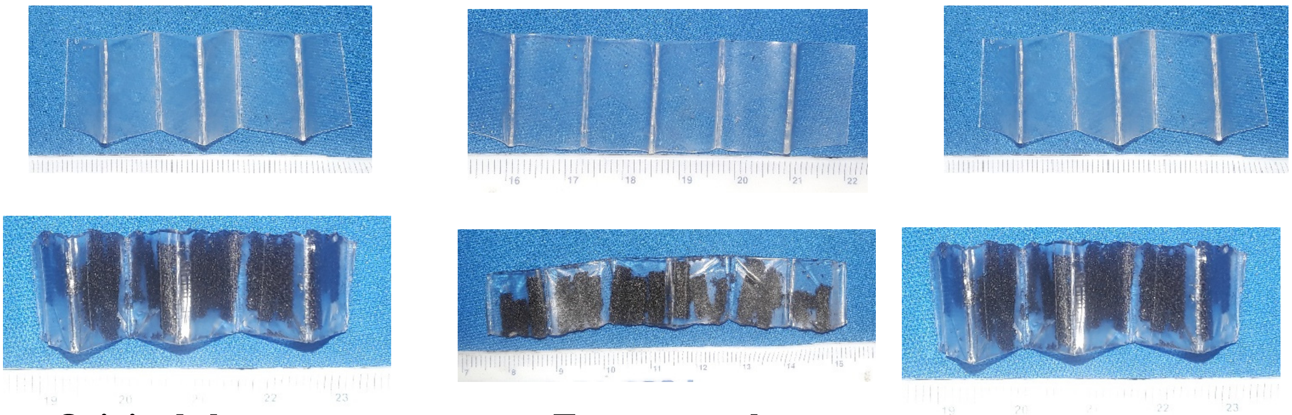

Figure S5. a) Images of epoxy SMP fabricated into accordion-like shape, stretched out into temporary shape and restored to its original shape under the influence of ***hot air***; b) Images of GrF-epoxy SMP made into accordion-like shape, deformed into temporary shape and recovered to its original shape when triggered by hot air
